# Identifying future partner agencies: helping Brazos Valley Food Bank in the fight against food insecurity

**DOI:** 10.1007/s43762-022-00064-9

**Published:** 2022-10-09

**Authors:** Sanni Saari, Ying Li, Shannon Avila, Ebony Knight

**Affiliations:** 1 College Station High School, 4002 Victoria Avenue, College Station, TX 77845 USA; 2 Department of Information and Operations Management, Mays Business School, Texas A&M University, College Station, TX 77843 USA; 3 Brazos Valley Food Bank, 1501 Independence Avenue, Bryan, TX 77803 USA

**Keywords:** Food insecurity, Geographic information systems, Multi-criteria decision making, Food bank, Food desert

## Abstract

Brazos Valley Food Bank (BVFB) is a non-profit organization in the Bryan-College Station area of Texas. It distributes food supplies through partner agencies and special programs to eradicate hunger in Brazos Valley. However, a big gap exists between the meals distributed by BVFB and the size of the food-insecure population. This research is motivated by BVFB’s desire to reach more people by recruiting more sustainable partner agencies. We used Geographic Information Systems (GIS) to map food desert areas lacking access to nutritious food. We combined expert knowledge with multi-criteria decision-making (MCDM) to address the challenges and time consumption of manually identifying sustainable partner agencies for local food delivery. We identified evaluation criteria for all agencies based on BVFB managers’ preferences using a qualitative approach, and then applied three quantitative decision-making models: the Weighted Sum Model (WSM), the Technique for Order Preference by Similarity to Ideal Solution (TOPSIS), and the Multi-criteria Optimization and Compromise Solution (VIKOR) models to obtain ranking results. We compared the quantitative models’ rankings to BVFB managers’ manual choices and discussed the impacts of our research. The key innovation of the research is to develop a mixed method by combining expert knowledge with mathematical decision models and GIS to support spatial decision making in food distribution. Although our results were specific to BVFB, these procedures can be applied to food banks in general. Future studies include finetuning our models to measure and address human biases, wider applications and more data collections.

## Introduction

Food insecurity is a serious problem that millions of Americans are facing. In 2020, 10.5% of American households were food-insecure at least sometime during the year (USDA, 2021). Feeding America estimated that 45 million people, including 15 million children, may have experienced food insecurity in 2020 (Feeding America, 2021a). In the United States, food pantries became an important food source for economically marginalized families (Lohnes & Wilson, 2018; Robaina & Martin, 2013). The COVID-19 pandemic made the dependence even bigger (Feeding America, 2021b). Food banks are non-profit organizations that function like distribution centers of food. Upon receiving food supplies from various sources, food banks sort them out, consolidate them, and turn them into packages (bulk or individual) that are ready to be sent out. Food banks usually distribute their food supplies through partner agencies (e.g., churches, shelters, mobile pantries) or special programs instead of directly to food-insecure populations to amplify the network across various service areas (Brazos Valley Foodbank, 2022; Houston Food Bank, 2022). Those partner organizations are responsible for local food distribution.

Food banks play a crucial role in the fight against food insecurity in the United States. Efficient food bank logistics and equitable food distribution to food-insecure communities are important topics for operations literature. Existing research efforts focus on routing and scheduling issues associated with food collection and distribution to and from food banks (Balcik et al., 2014; Davis et al., 2014; Lien et al., 2014; Solak et al., 2014; Reihaneh & Ghoniem, 2018). Some research also looks for algorithms and insight that help with equitable food distribution across counties (Sengul Orgut et al., 2016, 2017, 2018; Alkaabneh et al., 2021; Hasnain et al., 2021; Islam & Ivy, 2021; Stauffer et al., 2022). These studies cover many areas of food bank operations but assume that a food bank worked with a fixed set of partner agencies. The assumption allows this stream of studies to delve into operational details regarding efficiencies and equitable distributions within existent networks of partner agencies for food banks. In the reality, the number and capability of partner agencies may not be stable. This is due to the nature of the partner agencies. They are non-profit organizations and many of them, for example, food pantries, soup kitchens, shelters, etc. are run by volunteers. As such, the stability of a partner agency is not guaranteed. Since food banks’ distributions are mostly accomplished via partner agencies and food banks are evaluated by Feeding America by their distribution equity (Stauffer et al., 2022), food banks have an incentive to continuously maintain and expand its network of partner agencies to reach enough or more population with food insecurity. The number and quality of their partner agencies can either strengthen or weaken food banks’ service quality and quantity. Our research proposes analytical methods to help food banks find suitable partner agencies by examining the criteria that a food bank looks at when making the decision to reach out to a new partner agency. We used Brazos Valley Food Bank (BVFB) as an example to illustrate our proposed methodologies. We took a two-fold approach with Geographic Information Systems (GIS) applications and Multi-Criteria Decision Making (MCDM), respectively.

GIS plays an essential role in mapping food deserts for planning purposes, such as deciding which areas to target food services to (Feeding America, 2021a; Bradley & Vitous, 2019). MCDM is, in theory, an effective way to support decision-making, especially when dealing with conflicting decision objectives (Malczewski, 1999, 2006). In the past, MCDM has been combined with GIS to solve various spatial decision-making problems (Malczewski, 1999; Zhang et al., 2014; Zhang, Hu, et al., 2018; Zhang, Demsǎ, et al., 2018), like routing problems for food banks (Schneider & Nurre, 2019). An MCDM nonlinear programming approach has also been developed to improve groundwater, energy, and food nexus management that optimally allocates resources to food production (Radmehr et al., 2021). Since food banks consider multiple criteria when ranking candidate partner agencies and some of the criteria conflict with each other (e.g., higher poverty vs. better transportation access in the neighborhood), MCDM is an appropriate tool to facilitate food banks’ decision-making.

The key innovation of the research is to develop a mixed method by combining expert knowledge with mathematical decision models and GIS to support spatial decision making in food distribution. Using GIS, we mapped the food deserts in Brazos County, Texas, to identify areas where outreach efforts are most needed. We interviewed BVFB’s managers about how important each criterion (e.g., number of parking spaces, its accessibility from roads) is to them and integrated this information as a weight for each criterion in the MCDM models. We applied three models: the Weighted Sum Model (WSM), Technique for Order Preference by Similarity to Ideal Solution (TOPSIS), and Multi-criteria Optimization and Compromise Solution model (VIKOR) to rank BVFB’s potential partner agencies in the county in terms of its suitability for working as a food distributor. After that, we compared the results from these models to BVFB managers’ top choices to observe the differences between human decisions and math-based decision recommendations. Although our results were specific to BVFB, these procedures can be applied to all food banks in other areas.

Section 2 of this paper provides background information on BVFB’s operations and clarifies the organization’s definition of a food desert and other related terms. Section 3 introduces the data and key methodologies used to map food-insecure regions and develop the MCDM models to evaluate future partner agencies. In Section 4, we discuss the results of our analysis. Building upon our research efforts, Section 5 highlights managerial insights and the potential impact of this research on the effectiveness and sustainability of food banks’ outreaching efforts.

## Background

Our research team is composed of domain experts with institutional knowledge of BVFB as well as academic researchers. Section 2.1 provides relevant details about BVFB, and Section 2.2 discusses the definition of a food desert and related concepts.

### Introduction to BVFB’ operations and challenges

BVFB is a non-profit organization in the Bryan-College Station area of Texas. Through its partner organizations, BVFB serves Brazos Valley, which comprises six counties in central Texas: Brazos, Burleson, Grimes, Madison, Robertson, and Washington. Since 1985, BVFB has been working toward its vision of “a hunger-free Brazos Valley.” In 2017-2018, BVFB distributed 6,858,438 pounds of food, the equivalent of 5,621,670 meals (Brazos Valley Foodbank, 2022). Although this was an outstanding achievement, the number of delivered meals was insufficient to serve all food-insecure people in Brazos Valley. According to Feeding America, Brazos County alone had 43,590 food-insecure people (Feeding America, 2021a). If each of them was provided one meal per day, BVFB would have to deliver 15 million meals annually. The gap presented both challenges and opportunities for BVFB to expand its outreach efforts.

**Fig. 1:**
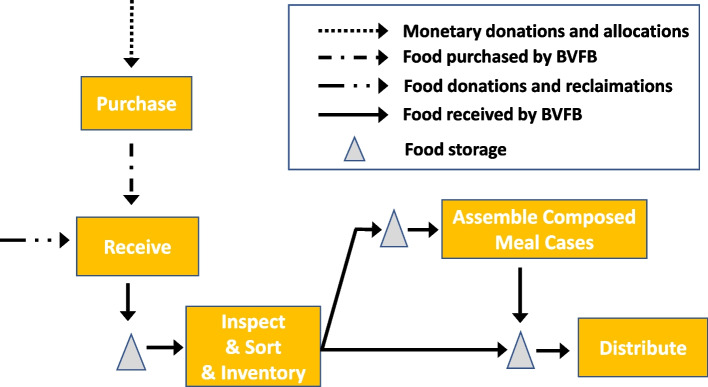
BVFB’s operation flow chart

To illustrate BVFB’s process of food collection and distribution, Fig. 1 shows BVFB’s operation flow chart to demonstrate how BVFB turns inputs (donations and purchases) into outputs (food ready to be handed to the community). These descriptions of BVFB’s operations and challenges were collected through interactions with the BVFB practitioners on our team. The BVFB practitioners identified two types of inputs: food and money. Using monetary donations and government funds, BVFB buys high-demand and nutritious food items to supplement what is not available from food donations and reclamations (Brazos Valley Foodbank, 2022). Food donations come from various sources: federal agencies, state agencies, local communities, private sectors, etc. These sources account for about 80% of BVFB’s food supply. Damaged but consumable food from grocery store chains accounts for another 10%. The last 10% of food supply comes from BVFB’s own purchases.

BVFB stores, inspects, and sorts its food while keeping an inventory record. BVFB’s demand comes from partner agencies and special programs. Partner agencies are non-profit organizations (e.g., churches) that run food pantries and on-site feeding programs. They place orders (mostly involving bulk food) to BVFB regularly. Special programs, including backpack programs, school-based food pantries, and senior outreach programs, need individual meal packages that can be distributed to individuals without any further assembly or packing.

Consequently, BVFB either distributes its food supply in bulk or as composed meal packages, which are designed by BVFB’s program staff and reviewed by their nutrition team. The assembly of composed meal packages requires bulk food to be unpacked, sorted and repacked. Local volunteers and BVFB’s staff usually perform these in-house tasks. Although BVFB has a steady supply of food, the types of food and the amount of each type it receives varies a great deal from month to month and from year to year. Furthermore, BVFB does not dictate the type and quantity of food ordered by its partner agencies or the number of meal packages needed by its special programs. Although supply and demand variability pose challenges to BVFB, it is still able to fulfill orders from partner agencies and special programs through dynamic food purchasing decisions and designing nutritious meal packages adjusted to varying food supply. The most pressing challenge BVFB faces is reaching more people given the discrepancy between the amount of food BVFB is distributing and the amount of food needed by the low-income population in Brazos Valley.

Without direct contact with the food-insecure population, BVFB hopes to reach people by sustainably increasing the number of partner agencies in each county of Brazos Valley since partner agencies are the most important outreach channel for BVFB. Hence, we focus on BVFB’s network of partner agencies and hope to provide insight that can guide BVFB’s efforts to recruit more sustainable partner agencies.

Building a network of sustainable partner agencies is a tedious and time-consuming process. For each applicant, BVFB conducts a vetting process and evaluates the applicant based on a set of criteria. For example, our BVFB domain experts pointed out that they would prefer a partner agency to serve low-income communities and to have sustainable operations, ease of access to major roads, a sufficient number of parking slots, and large open spaces for delivering the food. BVFB also looks at sustainability-related factors like years in operation and regular opening hours. Given many criteria, it is difficult to identify an organization that satisfies all of them. Often, BVFB has to prioritize different criteria in order to pick the partner agencies that best fit BVFB’s mission. An attractive potential partner based on some factors can be undesirable based on others. How should BVFB prioritize potential partner agencies’ applications? Which applicants would be a valuable addition to BVFB’s network of partner agencies? A map visualization of underserved locations as well as a systematic approach that ranks potential partner agencies based on BVFB’s standards would be helpful. This research provides science-driven solutions that can support BVFB in its efforts to build a network of sustainable partner agencies.

### Definition of food desert and related concepts

The term “food desert” has been used widely to refer to areas that lack access to affordable and nutritious food. Identifying those areas is a crucial step for BVFB to allocate resources effectively as it expands its outreach efforts. The concept of food deserts first appeared when USDA researchers identified areas “with low levels of access to retail outlets selling healthy and affordable foods” (Breyer & Voss-Andreae, 2013; Ploeg et al., 2011). A natural interpretation of “retail outlets selling healthy and affordable foods” would be grocery stores. They considered a food desert to exist when a supermarket is not present within a mile of an urban population center or within 10 miles of a rural population center (American Nutrition Association, 2022). Although this definition of “food desert” has been accepted by many organizations, it has faced criticism for making false assumptions about the food habits of economically marginalized families (Guthman, 2011). A literature review indicated that most economically marginalized families left their neighborhoods to shop at faraway supermarkets because they prioritized price over convenience (Alkon et al., 2013). This finding implied that locations in the vicinity of supermarkets could be food deserts as well. This literature also showed that those economically marginalized families emphasized the quality of food rather than the quantity they could obtain. Refined concepts like “food swamp” and “food mirage” were sometimes used to compensate for what is missing in the original definition of “food desert” (Hager et al., 2017; Haskell, 2021; Lindner, 2021). While a food swamp refers to an urban environment where fast food and processed food overshadow nutritious food, a food mirage refers to a neighborhood where financial conditions limit access to available nutritious food.

The discussions on food deserts, food swamps, and food mirages motivated us to think carefully about how to identify areas that lack access to nutritious food for BVFB. As we mapped those areas, two types of food sources were taken into consideration for a population facing food insecurity: 1) grocery stores; 2) food pantries. The choice of grocery stores was consistent with the original and widely used definition of food desert (Guthman, 2011). The choice of food pantries was inspired by the definition of food mirage, which was caused by a lack of financial means.

## Methodology and data

The data used in this project comes from facility data (e.g., churches, shelters, grocery store locations) collected using google maps, publicly available census data, and the institutional knowledge of the practitioners on our team. We describe the methodologies and their corresponding data in three subsections below. Among the six counties BVFB serves in Central Texas, Brazos County has the highest number of people facing food insecurity: 43,590 (Feeding America, 2021a). For this reason, we used Brazos County as the case study area for analysis.

### Use of GIS in mapping the food insecure regions

As mentioned, we take into account two types of food sources (grocery stores and food pantries) in this research. We mapped locations lacking either of these two food sources using GIS. Our first step was to identify all food source locations (grocery stores and food pantries) on the map of Brazos County. Using Google Maps, we manually found the addresses of all grocery stores and food pantries and used GIS geocoding services to manually geocode all locations onto a map.

Our second step was to map the area that each food source serves. We adopted the convention of using one mile as a tolerable walking distance and considered any area within the radius of a mile of a food source as its service area. The complication in mapping was that different food source locations have overlapping service areas. For a clearer visual result, we plotted service areas of food source locations that were close to each as a combined region. In order to do so, we applied Dijkstra’s algorithm to traverse networks. Dijkstra’s algorithm is known to solve the single-source shortest paths for a given graph with nonnegative edge weights (Dijkstra, 1959). In our application, the algorithm returned a subset of connected edge features that were within a one-mile walking distance from a food source location. Subsequently, we input the edges into a triangulated irregular network (TIN) data structure (Peucker et al., 1978), where the network distances, along with the edges, served as the heights of the locations inside the TIN. Locations not traversed were put in with a much larger height value. An area-generation routine was used with the TIN to carve out regions encompassing areas in between the specified break values. As such, we could neatly plot the areas served by all food source locations on the map of Brazos County (see figures in Section 4.1 for details).

To help visualize poverty levels, we represented all census tracts in Brazos County on the map. The census tract level poverty data were obtained using 2016- 2020 American Community Survey 5 years data (U.S. Census Bureau, 2022b). In addition to the existing food pantries and grocery stores, we also plotted the future partner agencies and their reachable regions (one-mile distance) from the grocery stores and existing food pantries.

### MCDM decision analysis

MCDM is a mathematical decision-making framework in which several decision criteria are combined to meet one or several objectives that support decision-making (Triantaphyllou et al., 1998). In an MCDM model, a set of m alternatives should be given and denoted as A_1_, A_2_, A_3_, …, A_m_. According to policies governing food banks in general, all partner agencies of food banks must be approved by non-profit organizations (e.g., shelters, churches, and soup kitchens) and meet a set of minimum standards. Through extensive and careful google searches, we found 17 organizations in Brazos County that satisfy the minimum requirements to become a partner agency for BVFB. Those 17 organizations were considered the alternatives as we applied MCDM. In the interest of confidentiality, we used letters to label those organizations when presenting our results in Section 4. Based on the institutional knowledge of the practitioners on our team, we identified ten key evaluation criteria that are important for BVFB’s selection of partner agencies. Those factors are listed in Table 1. In an MCDM model, a set of n decision criteria should also be given but denoted as C_1_, C_2_, C_3_, …, C_n_.

**Table 1:** The description and weight of the criteria

	Criterion	Description	Weight
1	Poverty condition	Percentage of population under poverty in the same census tract	90
2	Opening hours	Number of opening hours per day	70
3	Population groups	Number of population groups the organization is serving	70
4	Years in operations	Number of years the organization has been in operations	60
5	Facebook likes	Number of likes on the organization’s Facebook page	50
6	Road access	Accessibility to major roads (on a scale of 1 to 3 with 3 being the best)	50
7	Yard condition	Number of people the yard can hold during food distribution	50
8	Existing food pantries	Number of existing food pantries within 1-mile walking distance	40
9	Facebook check-ins	Number of people checked-in on the organization’s Facebook page	40
10	Parking condition	Number of parking slots	30

We included existing food pantries as a criterion but not existing supermarkets. A higher number of existing food pantries in the neighborhood indicated less demand for establishing a new service. The locations of existing supermarkets do not affect BVFB’s evaluation of future partner agencies since we consider the low-income families who rely on food pantries rather than buying food from a grocery store. We measured accessibility to potential partner agencies via criteria like parking conditions, open space, and road access. We included poverty condition as an important indicator since the goal of the service is to provide food to more food-insecure people. Furthermore, the sustainability of a potential partner agency is important to BVFB’s long-term outreaching efforts. Therefore, we included a set of sustainability-related indicators such as serving groups, Facebook likes, Facebook check-ins, opening hours, and years in operation. The descriptions of the ten evaluation criteria are presented in Table 1.

Most multi-criteria methods require the definition of quantitative weights for the criteria in order to assess the relative importance of the different criteria. The Simple Multi-attribute Rating Technique (SMART) weighting is a process of rating alternatives and weighting criteria (Olson, 1997; Odu, 2019). The criteria are ranked by their importance from least to most important from a scale of 10-100. After that, the criteria weight can be calculated by normalizing the sum of the points to one. Saaty (1977) proposed a numerical scale from 1 to 9 to transform qualitative data into quantitative data by including the ideas that describe equal importance (e.g., with intensity of importance score 1) and absolute importance (e.g., with intensity of importance score 9). We combined ideas from Saaty (1977) and SMART, so the decision maker can assign a weight to each criterion from a scale of 10-100 points without having to give any criterion an absolute 100 that the SMART method requires. With this mixed approach, alternatives can also receive an equal weight if they contribute equally to the suitability of the partner organization.

In the next step, the MCDM analysis requires decision-makers to collect or determine the performance value a_ij_ (for i = 1,2,3, …, m and j = 1, 2, 3, …, n) for each alternative and each criterion. Based on the performance matrix and weights, various techniques are used in different MCDM models to rank all the alternatives. For example, a more significant value percentage in poverty is preferred to build a new pantry. On the other hand, the higher number of existing food pantries in the region is less preferred than building a new service. The data value and weights were normalized to reflect such logic. Usually, MCDM models normalize the weights so that they add up to one.

The following actions describe our efforts to collect data on the performance values a_ij_. As described above, we have 17 alternatives and ten evaluation criteria. We used Google Maps Street View to observe the parking and yard (potential distribution space) conditions of the 17 organizations of interest. The percentage of the population under poverty was estimated using Census data at the Census Tract level (US Census Bureau, 2022a). We also visited the Facebook and official website of each of the 17 organizations to find the values for criteria such as the number of Facebook likes (which reflect the popularity of an organization), opening hours, the number of population groups served, and the years in operation. We fit the performance values in three types of MCDM models (WSM, TOPSIS, and VIKOR) to rank the suitability of the 17 potential partner agencies.

WSM is the earliest and likely the most widely used MCDM method (Fishburn, 1967), while TOPSIS and VIKOR are other methods that are often compared to each other and with WSM (Hwang & Yoon, 1981; Opricovic & Tzeng, 2004; Ploskas & Papathanasiou, 2019).

#### Weighted sum model (WSM)

If there are m alternatives and n criteria, the best alternative is the one that satisfies (in a maximization case) the following expression (Fishburn, 1967):

1 $$A={\mathrm{max}}\ {\sum\limits_{j=1}^n\kern0.5em {a}_{ij}{w}_j,}\ for\ i=1,\dots, m$$

In the equation, n is the number of decision criteria, a_ij_ is the performance value of the i^th^ alternative in terms of the j^th^ criterion, and w_j_ is the weight or importance of the j^th^ criterion. The notation A represents the highest WSM score, which is also the WSM score associated with the best alternative. Note that a WSM score is the weighted sum of performance values associated with an alternative.

#### Technique for order preference by similarity to ideal solution (TOPSIS)

The TOPSIS method is a multi-criteria decision analysis method which is based on the concept that the best alternative should have the shortest geometric distance from positive ideal solution and longest geometric distance from the negative ideal solution (Hwang & Yoon, 1981; Behzadian et al., 2012; Zhang, Demsǎ, et al., 2018).

TOPSIS employs the following procedure:

1. Create an evaluation matrix consisting of m alternatives and n criteria with the intersection of each alternative and criteria given as *a*_*ij*_. After that, calculate normalized rating for each element in the decision matrix.
2. Calculate weighted normalized ratings v_ij_ using the formula:

2 $${v}_{ij}={w}_j{a}_{ij},i=1,\dots m;j=1,\dots, n.$$

3) Calculate positive ideal A^*^ and negative ideal A^−^ solutions in terms of the weighted normalized values, as shown below:

3 $${A}^{\ast }=\left\{{v}_1^{\ast },{v}_2^{\ast },\dots, {v}_j^{\ast },\dots, {v}_n^{\ast}\right\}=\left\{\left({\mathit{\max}}_i{v}_{ij}|j\in {J}_1\right)|i=1,\dots m\right\}$$

4 $${A}^{-}=\left\{{v}_1^{-},{v}_2^{-},\dots, {v}_j^{-},\dots, {v}_n^{-}\right\}=\left\{\left({\mathit{\min}}_i{v}_{ij}|j\in {J}_2\right)|i=1,\dots m\right\}$$

where J_1_ is a set of benefit attributes and J_2_ is a set of cost attributes.

4) Calculate separation measures ${S}_i^{\ast }$ and ${S}_i^{-}$. The separation between alternatives can be measured by n dimensional Euclidean distance. The separation of each alternative from the positive ideal solution is given by

5 $${S}_i^{\ast }=\sqrt{\sum_{j=1}^n\kern0.5em {\left(\Big({v}_{ij}-{v}_j^{\ast}\right)}^2},i=1,\dots, m$$

Similarly, the separation from the negative ideal solution is given by

6 $${S}_i^{-}=\sqrt{\sum_{j=1}^n\kern0.5em {\left(\Big({v}_{ij}-{v}_j^{-}\right)}^2},i=1,\dots, m$$

5) Calculate ${C}_i^{\ast }$, which measures similarity to the ideal solution for alternative m.

7 $${C}_i^{\ast }=\frac{S_i^{-}}{S_i^{\ast }+{S}_i^{-}}\ i=1,\dots m$$

with $0\le {C}_i^{\ast}\le 1$ where ${C}_i^{\ast }=0$ when *A*^∗^ = *A*^−^ and ${C}_i^{\ast }=1$ when *A*_*i*_ = *A*^∗^

6) Choose an alternative with maximum ${C}_i^{\ast }$ or rank the alternatives according to ${C}_i^{\ast }$ in descending order.

#### Multi-criteria optimization and compromise solution (VIKOR) model

VIKOR method has gained a popularity among the decision-making community (Opricovic, 1998; Opricovic & Tzeng, 2004). The basic concept of VIKOR method lies in defining the positive and the negative ideal points in the solution space. For instance, more Facebook likes is ideal while having more existing food pantries in the same 1-mile area is not ideal. VIKOR focuses on ranking and selecting from a finite set of feasible alternatives in presence of conflicting criteria. The mathematical formulation of the VIKOR method is illustrated below.

Let’s assume that we have a decision matrix with *m* alternatives and *n* criteria, and the matrix is represented as *X* = *f*_*ij*_(*A*_*i*_)_*m* × *n*_.

1. Determine the best ${f}_i^{\ast }$ and worst ${f}_i^{-}$ values for each criterion. Use Eq. (8) for profit criteria and Eq. (9) for cost criteria.

8 $${f}_j^{\ast }=\mathit{\operatorname{Max}}\ i\ {f}_{ij},{f}_j^{-}=\mathit{\operatorname{Min}}\ i\ {f}_{ij}$$

9 $${f}_j^{\ast }=\mathit{\operatorname{Min}}\ i\ {f}_{ij},{f}_j^{-}=\mathit{\operatorname{Max}}\ i\ {f}_{ij}$$

2) Compute the values of Si and Ri using the following equations:

10 $${s}_i=\sum\limits_{j=1}^N\ {w}_j\left[{f}_j^{\ast }-{f}_{ij}\right]/\left[{f}_j^{\ast }-{f}_j^{-}\right]$$

11 $${R}_i=\mathit{\operatorname{Max}}\ j\ \Big[{w}_j\left[{f}_j^{\ast }-{f}_{ij}\right]/\left[{f}_j^{\ast }-{f}_j^{-}\right],j=1,2,\dots, M$$

The beneficial criteria require higher values, so Eq. (10) is appliable. But non-beneficial criteria require lower values, so the term [(*f*_*ij*_)_*max*_ − *f*_*ij*_] in Eq. (10) is to be replaced by [*f*_*ij*_ − (*f*_*ij*_)_*min*_]. Hence, for non-beneficial criteria, Eq. (11) can be reformulated as:

12 $${s}_i=\sum\limits_{j=1}^N\ {w}_j\left[{f}_{ij}-{\left({f}_{ij}\right)}_{min}\right]/\left[{\left({f}_{ij}\right)}_{max}-{\left({f}_{ij}\right)}_{min}\right]$$

3) Calculate the Q_i_ value.

13 $${Q}_i=v\left(\left({s}_i-{s}_{i-\mathit{\min}}\right)/\left({s}_{i-\mathit{\max}}-{s}_{i-\mathit{\min}}\right)\right)+\left(1-v\right)\left(\left({R}_i-{R}_{i-\mathit{\min}}\right)/\left({R}_{i-\mathit{\max}}-{R}_{i-\mathit{\min}}\right)\right),$$

where *s*_*i* − *max*_ and *s*_*i* − *min*_ are the maximum and minimum values of *s*_*i*_ respectively, and *R*_*i* − *max*_ and *R*_*i* − *min*_ are the maximum and minimum values of *R*_*i*_ respectively. Variable *v* is introduced as weight of the strategy of “the majority of attributes” (or “the maximum group utility”). Its value lies between 0 and 1. Usually, the value of *v* as 0.5 is preferred. The compromise can be selected with “voting by majority” (v > 0.5), with “consensus” (v = 0.5) or with “veto” (v < 0.5). More specifically, when *v* equals unity, it represents a decision-making process that can use the strategy of maximizing group utility, whereas when *v* is zero, it represents a process that can use a minimum individual regret strategy that is found among maximum individual regrets/gaps of lower-level criteria for each alternative. The value of *v* affects the ranking of the alternatives and is usually determined externally by the decision expert.

4) Rank the alternatives, sorting by values S, R, Q in ascending order.
5) Normally, S, R, Q ranking list should be used to propose the compromise solution as shown in Opricovic (1998) and Opricovic & Tzeng (2004). In this paper, we used the Q ranking list.

### Use of expert knowledge in models’ validation

The evaluation criteria and weights of the MCDM models were selected based on the BVFB domain experts’ intuitional knowledge. The models implemented different procedures to arrive at their ranking results. The implementations were, naturally, not influenced by human experience or judgment. In the real world, food banks did not routinely apply MCDM models when ranking potential partner agencies. Instead, experiences and intuitions played an important role when BVFB recruited new partner agencies. To check the practicality of our quantitative models, we used experience-based validation.

While the researchers on our team fed performance values to the MCDM models, the BVFB practitioners on the team examined the same performance values and picked their top three out of the 17 alternatives before seeing any results generated from the MCDM models. Then the whole team examined the rankings from the MCDM models and the top choices of the practitioners together. A comparison of the two types of results generated interesting observations, which are presented in Section 4.3.

## Results

### Mapping food deserts

**Fig. 2:**
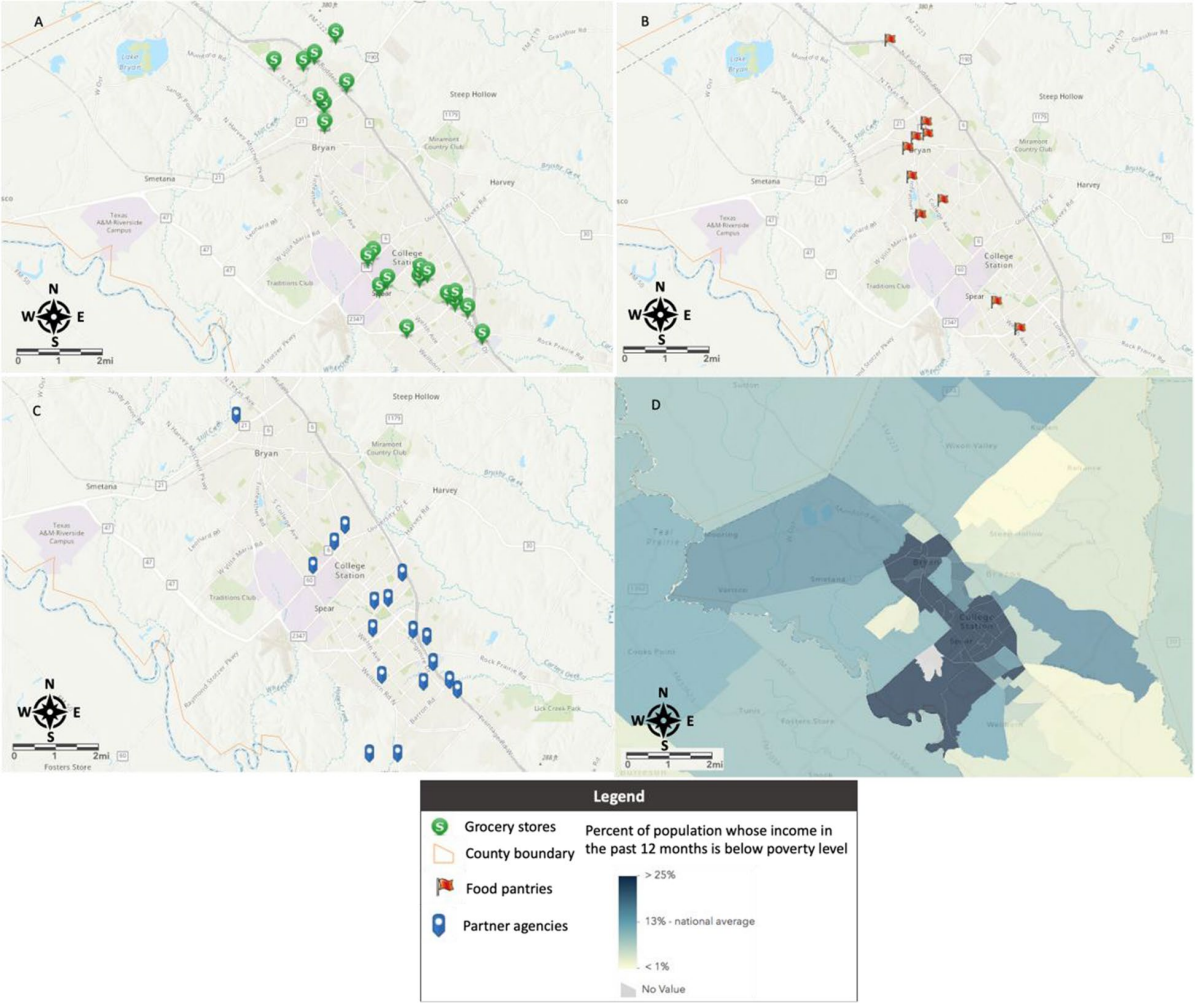
Mapping food resources and poverty in Brazos County

**Fig. 3:**
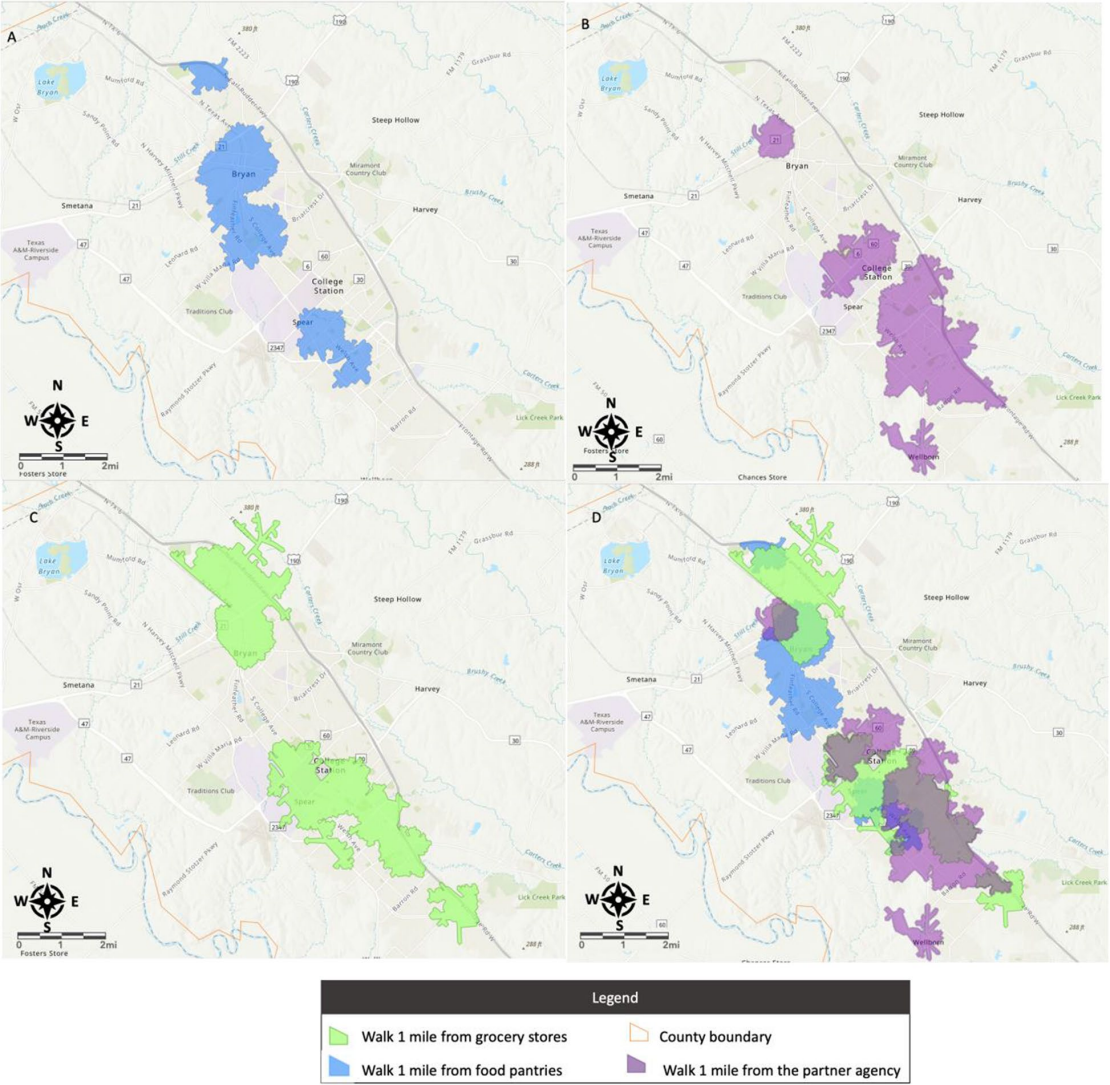
Mapping the food deserts

We plotted food resources including grocery stores, existing food pantries, and future partner agencies in Fig. 2. A food desert map for Brazos County is presented in Fig. 3. The food desert areas refer to the regions outside the purple, green, and blue buffers on the map, which have limited access (e.g., more than 1 mile of walking distance) to grocery stores and existing and future food pantries. The food desert map will help BVFB managers picture the food-insecure locations and identify the places that may need more services in Brazos County. In Figs. 2 and 3, we plotted most data layers into separate maps to produce better visualization results. Figure 3d also provides overlayed map to show the overall picture of the food deserts.

We used the 2016-2020 American Community Survey 5 years data at tract level managed by the U.S. Census Bureau to create the poverty map. The darker color represents the higher percentage (e.g., over 25%) of the population whose income in the past 12 months is below the poverty level (U.S. Census Bureau, 2022b). The areas with accessibility (one mile walking distance) to grocery stores and existing food pantries were plotted in purple and blue, respectively. Geo-visualization techniques and Dijkstra’s algorithm (see Section 3.1) are used to compute the reachable area from the food sources including existing grocery and food pantries and future food delivery locations. The blue drop pins represent the locations of the potential partner agencies we examined for BVFB. Purple regions represent the expanded food delivery regions by partners with each organization. The region created using the Dijkstra’s algorithm around each organization of interest (the blue drop pin) to help visualize their coverage and possible overlap with existing food pantries in its one-mile vicinity.

The map shows that there are overlaps - although not many - between purple and blue regions. As mentioned earlier, BVFB does not consider grocery stores as food sources for food-insecure populations. In this case, making outreach decisions based on the original food desert definition that used grocery stores as food sources can be misleading. The map also shows that the potential candidates (dark purple regions Fig. 3) of the partner agencies are mostly in areas lacking access to food pantries, meaning that their operation could shrink the size of food deserts in Brazos County.

### The results from MCDM analysis

Valuing confidentiality, we replaced the real names of our 17 organizations with labels A, B, C, etc., in this paper. While Organization A through P are churches, Organization Q is a charity that provides shelter to families with children. We noted that the majority of existing food pantry programs in Brazos Valley were run by churches. Table 2 presents the performance values we collected and/or estimated. For ease of presentation, we use the number labels of the criteria, instead of the names of the criteria, in Table 2. The list of the criteria and their corresponding number labels are presented in Table 1 earlier.

**Table 2:** Performance values of the 17 organizations of interest (i.e. alternatives)

*Organizations*	*Criteria*									
	*1*	*2*	*3*	*4*	*5*	*6*	*7*	*8*	*9*	*10*
*A*	30%	8	3	10	554	1	30	0	24	5
*B*	30%	7	3	622	727	1	10	0	8	2
*C*	2.9%	1	1	0	0	3	50	0	2	40
*D*	2.9%	12	3	977	454	3	50	0	7.5	100
*E*	14.8%	1	3	200	227	1	10	0	2	2
*F*	14.8%	1	3	103	1090	3	20	0	9	50
*G*	10%	12	3	500	568	3	100	0	9	20
*H*	7.7%	13	3	1305	498	3	100	0	3	40
*I*	13%	14	3	368	529	3	40	0	1.5	20
*J*	43%	13	3	3000	6292	3	100	1	2	50
*K*	51.6%	11	3	535	1577	1	30	0	1	50
*L*	38%	11	3	1240	295	1	30	0	9	30
*M*	57.6%	10	3	547	417	3	100	0	1	20
*N*	40%	12	3	606	1013	3	40	0	9	100
*O*	61.9%	13	3	4552	2524	3	10	0	9	6
*P*	40.6%	10	3	1575	866	3	15	0	2	20
*Q*	40.6%	12	3	65	715	1	15	3	8	30

We considered each potential partner agency as an alternative to apply in the WSM, TOPSIS, and VIKOR methods. For example, in the WSM and TOPSIS methods, *a*_*ij*_ is the performance value (e.g., number of parking slots, opening hours per day, number of Facebook likes) of the i^th^ alternative in terms of the j^th^ criterion/indicator, and *w*_*j*_ refers to the weight of each indicator as shown in Table 1. We calculated the WSM score for each alternative (namely, potential partner agency) and ranked all of them based on their scores in descending order. Following the procedure described in Section 3.2.2, we calculated the separation measures (${S}_i^{\ast }$ and ${S}_i^{-}$) and similarity to the ideal solution (${C}_i^{\ast }$) for each alternative and ranked all alternatives based on their ${C}_i^{\ast }$ in descending order. The ranking results are reported in Table 3.

**Table 3:** Ranking results of the potential partner agencies using WSM, TOPSIS, and VIKOR

*Organization*	*WSM rank*	*TOPSIS rank*	*VIKOR rank*
*A*	10	6	4
*B*	15	12	8
*C*	17	14	17
*D*	7	4	15
*E*	16	15	16
*F*	13	3	14
*G*	5	2	11
*H*	6	7	13
*I*	12	9	12
*J*	1	16	3
*K*	9	13	10
*L*	11	10	5
*M*	4	11	6
*N*	3	1	1
*O*	2	5	2
*P*	8	8	9
*Q*	14	17	7

### Validation of the ranking results

We compared the results generated by each MCDM model. Results showed that the VIKOR model differed from the WSM and TOPSIS models in alternative B, D, G, H, L, and Q. For instance, organization B received low ranking results from WSM (ranking 15) and TOPSIS (ranking 12) models and received an 8 from the VIKOR method. A similar situation also applied to organization Q. The opposite situation happened to organization D; it was placed at the higher ranks of 7 and 4 respectively by WSM and TOPSIS but placed nearly last by VIKOR. Additionally, some striking differences appeared for TOPSIS. For instance, it gave the rank of 3 to organization F, while WSM and VIKOR gave it a 13 and 14, respectively. Organization J was ranked first by WSM and third by the VIKOR method but ranked low according to TOPSIS. Even though there is an existing food pantry in the vicinity of Organization J, the large size of its yard and the higher number of parking slots, as well as the high poverty level, make this organization a standout from the list of potential partner agencies. This organization is a church that has actively served all types of populations for over 13 years. Its many Facebook likes and check-ins reflect how active and vibrant it was during the past years. Aside from striking differences, WSM, TOPSIS, and VIKOR methods were consistent on their low-ranked alternatives: Organizations C and E, which are located in wealthy neighborhoods (2.9% poverty rate). Organization C serves only one ethnic group, which does not have active Facebook activities. Organization E does not seem to be active at all; its opening hours are short, the location has a small yard and parking lot, and it has very little Facebook activity. Organizations N and O generally received good high-ranking scores from all three models.

Another aspect that we analyzed is how closely model-made decisions match with the instinct of the practitioners of this research team, the food bank managers. The practitioners examined Table 2, which included performance values collected on the 17 potential partner agencies and picked their top three candidates before learning the results from MCDM models; the practitioners were aware of the real names of those organizations.

They gave organizations O and K the top score and M a three; organizations K and O are tied as No. 1 according to the practitioners. They liked the overall portfolio of both Organization K and O but recognized that the location of Organization O could cause access problems for nearby residents. They believed that the type of distribution that would be best suited to Organization K and O might look different than a drive-through or mass distribution. There are many ways food pantries can distribute food. Drive-through and mass distribution are the more efficient and popular approaches. However, these two widely applied approaches imply that all clients receive the same food (in pre-packed bags or boxes). In contrast, by-appointment distributions generally serve fewer people but allow clients to choose the food best suited to the needs of their households. Our practitioners ranked Organization M as the third place because it would provide good visibility due to its physical location in the region, which was not considered a criterion in our models. In addition, the practitioners found Organization C a possible valuable addition to BVFB’s network because of the particular ethnicity of the population it served. However, organization C has problems with sustainability and opening hours according to our models. From these three organizations, only O matches the high score the models gave it, but M mainly was given a medium score from WSM and VIKOR, and K was given a relatively low score from all three models. This finding suggests that MCDM may consider more details than humans with pure instinct but may also fail to consider aspects that aren’t explicitly modelled.

## Conclusions and discussion

Food banks are essential in the fight against food insecurity in the U.S. In order to understand how foodbanks function and the needs of BVFB in particular, we reviewed relevant literature, spent several months volunteering at BVFB, and collaborated with practitioners with insight of BVFB. The task of meeting supply with demand is a tall order for food banks to execute because they have limited control over supply and demand. BVFB has done a great job with its resources and believed that the best way to achieve its goal of a “hunger-free” Brazos Valley is to recruit more sustainable partner agencies.

We applied a GIS algorithm and geo-visualization techniques to illustrate the impacts of different definitions of “areas lacking access to nutritious food” on mapping for Brazos County. Outcomes show that the potential partner agencies we picked for BVFB mainly were in areas lacking access to food pantries instead of in areas lacking grocery stores. The techniques we used for mapping can be later applied to other counties in Brazos Valley and other regions. Such maps visualize the areas in need of food pantries and can help food banks use their resources effectively in their outreach efforts.

The vetting process of a partner agency is tedious and time-consuming. BVFB examined several factors (e.g., sustainability of operations, physical conditions, and accessibility in low-income communities) when determining future partner agencies. We compared three MCDM models to develop a science-driven solution to help BVFB reach future partner agencies. Both TOPSIS and VIKOR methods are based on measures of distance from reference objects. Our study shows that the choice of selection methods plays a crucial role in ranking partner agencies given multiple criteria. We chose not to use the Analytic Hierarchy Process (AHP) (Saaty, 2004) method because of the complexity of conducting pair-wise comparison for ten evaluation criteria (e.g., 10 × 10 pair-wise comparison needs to be developed using the AHP method). When dealing with large evaluation criteria, WSM, TOPSIS, and VIKOR are more effective. Given the setting, we believe MCDM models are useful tools to support BVFB’s evaluation of future partner agencies. We illustrated how MCDM models could help with a case study on Brazos County by identifying 17 potential partner agencies in the county, creating a criteria table based on the knowledge of the practitioners on our team, collecting performance values, and applying our three MCDM models.

To validate the ranking results from the MCDM models, we compared those results to the top choices of the practitioners, which revealed both agreements and discrepancies. It is interesting to see how factors that are difficult to be articulated or measured affect the practitioners’ choices. For example, the visibility of a location, which was not a criterion in the MCDM models, significantly boosted an organization’s standing with the practitioners. Meanwhile, the low performance values on parking condition, yard space and opening hours, which were criteria considered by the MCDM models with non-trivial weights, did not hurt some other organizations’ standings with the practitioners because they were able to come up with a mitigating mode of food distribution that would circumvent those drawbacks. Nevertheless, we were excited about the collaboration between the researchers and practitioners who value the potentials of the MCDM models. These models are easy to adjust and can provide evaluations of future partner agencies. The comparisons between results of the MCDM models and the practitioners’ judgments support a more complete picture of how a food bank can advance its mission sustainably and effectively.

We recognize that the assignment of weights affects MCDM models’ outcomes. Relying on practitioners’ direct inputs allows us to build practical models. But human biases are inevitable under this approach. In addition, the criteria articulated by practitioners may not include the ones that only surface in special cases. We plan to address these biases or measure these biases in future studies. We also hope to collect more field data that can help us validate the criteria choices and weight assignments.

Although our results were specific to BVFB, these procedures can be applied to food banks in other geographic areas. In the future, we hope to apply our decision-making models to more food banks and find out how well they would be adapted.

## Data Availability

Data utilized in this research are public information.
